# Optical characterization of non-thermal plasma jet energy carriers for effective catalytic processing of industrial wastewaters

**DOI:** 10.1038/s41598-021-82019-4

**Published:** 2021-02-03

**Authors:** M. Y. Naz, S. Shukrullah, S. U. Rehman, Y. Khan, A. A. Al-Arainy, R. Meer

**Affiliations:** 1 Department of Physics, University of Agriculture, Faisalabad, 38040 Pakistan; 2 Department of Electrical Engineering, Namal Institute Mianwali, Mianwali, Pakistan; 3 College of Engineering, King Saud University, Arriyadh, 11437 Saudi Arabia

**Keywords:** Chemistry, Engineering, Materials science, Physics

## Abstract

An argon plasma jet was sustained in open air and characterized for its chemical composition. The optically characterized plasma jet was used to treat industrial wastewater containing mixed textile dyes and heavy metals. Since plasma jet produces UV-radiations, the photocatalytic TiO_2_ was used to enhance plasma treatment efficiency especially for degradation of dyes. Mixed anatase and rutile phases of TiO_2_ (5.2–8.5 nm) were produced through surfactant assisted sol–gel approach. The emission spectrum confirmed the presence of excited argon, OH, excited nitrogen, excited oxygen, ozone and nitric oxide in the plasma jet. The spectral lines of excited Ar, NO, O_3_, OH^−^, N_2_, ${\mathrm{N}}_{2}^{+}$, O, ${\mathrm{O}}_{2}^{+}$ and O^+^ species were observed at wavelength of 695–740 nm, 254.3 nm, 307.9 nm, 302–310 nm, 330–380 nm, 390–415 nm, 715.6 nm, 500–600 nm and 400–500 nm. These reactive species decompose the organic pollutants and separate the heavy metals from the water samples. The conductivity of plasma exposed water samples increased while pH and hardness decreased. The atomic absorption spectrophotometry analysis confirmed the presence of heavy metals in the samples, which were effectively removed through plasma treatment. Finally, the effect of plasma treatment on Staphylococcus aureus strains was more pronounced than *Escherichia coli* strains.

## Introduction

The contamination of water bodies is generally caused by the release of pollutants into groundwater or into streams, lakes, estuaries, rivers and oceans. The polluting substances degrade the water quality and natural functioning of ecosystems^1,2^. In developing countries, the coliforms, pesticides, toxic metals and industrial effluents are the major sources of surface and subsurface groundwater pollution. The disposal of industrial effluents and heavy metals in water bodies raises the human health concerns, poisons the wildlife, and damages the long-term ecosystem^3^. Toxins in industrial effluents promote the reproductive failure, immune suppression and acute poisoning^4^. The bacterial strains in water release toxins in digestive tracts, which cause nausea, watery diarrhea, vomiting, renal failure and dehydration. The harmful bacteria are removed from water through antimicrobial treatment. In developed countries, strict regulations are imposed on industrial and agricultural operations to minimize the contamination of water bodies. Different methods are also being deduced to prevent the flow of pollutants into the water bodies and to remove the pollutants from wastewater^5^. With conventional water treatment techniques, such as chlorination, coagulation, adsorption, ultra-sonication, etc., it is difficult to eliminate all the harmful contaminants from the water. Recently, some advanced oxidation techniques like irradiation of high energy electrons, oxidation using ozone, ionizing radiations exposure, carbon absorption, plasma exposure and sonolysis have been practiced for the treatment of contaminated waters^4^.

The non-thermal plasma jet is one of the best oxidation methods available for the treatment of polluted water. A non-thermal plasma jet is an electrically energized gas, which is produced by passing a gas through a strong electric field^6^. Instead of just gas heating, the bulk of electric field energy goes into the creation of energetic plasma species. These species include positive ions, electrons, negative ions, free radicals, electrically neutral gas atoms and or molecules and electromagnetic radiations. Being strong oxidizers, the plasma species strongly interact with the contaminated media and decompose the organic and inorganic compounds in the media. These plasma species also kill the bacterial endospores and vegetative cells. The highly energized photons, ozone, atomic oxygen, and free reactive oxygen radicals in the plasma damage the cells by charging the cell wall and reacting with macromolecules. Since membrane lipids at the cell surface are susceptible to the reactive oxygen species, the oxidized cytoplasmic membrane lipids release intracellular substances, which damage the cells. Moisan et al.^7^ proposed some mechanisms of inactivation of microbial spores under non-thermal plasma exposures. These mechanisms are based on interaction of ultraviolet radiations with spore surface and volatilization of surface compounds, damaging of DNA by ultraviolet irradiation, and erosion or etching of spore surface with reactive oxygen radials.

The reactive oxygen radials and nitrogen oxides in plasma jet not only kill the living organisms but decompose the organic and inorganic compounds as well^4,8^. If a suitable photocatalyst is added to the contaminated water, the plasma treatment of polluted water may be more effective. Titanium dioxide (TiO_2_) is a well-known photocatalyst. Under suitable light exposure, it converts the pesticides, polymers, surfactants, aliphatics, aromatics, herbicides and dyes into water, mineral acids and carbon monoxide^9^. TiO_2_ is a polymorphous material, which exists in anatase, rutile and brookite phases. All these phases show octahedral structures but differ in the arrangement of their octahedral units^10,11^. Anatase phase is the most prominent commercial phase of TiO_2_ due to its better stability and photocatalytic activity as compared to rutile and brookite phases^4^. TiO_2_ nanoparticles are also known for good surface acidity, good thermal and chemical stabilities and low toxicity potential^12^. Karami et al*.*^13^ and Wang et al*.*^14^ revealed that photocatalytic and semiconducting activities of anatase phased TiO_2_ mainly depends on crystal structure, crystallite size, shape, active surface area and overall morphology. The specific optical and structural properties of TiO_2_ nanostructures can be tailored through a deliberately chosen and well optimized method of synthesis. In many cases, sol–gel method is preferred over other methods when it comes to low cost production of nanomaterials, ceramics and glass. In this study, a sol–gel method was adopted to produce mixed anatase and rutile phases of TiO_2_ nanoparticles. The photoactive TiO_2_ was used to degrade the organic compounds in contaminated water under direct current plasma exposure.

## Materials and methods

### Preparation of TiO_2_ photocatalyst

TiO_2_ catalyst was produced by practicing a simple sol–gel technique. Hydrochloric acid was used as a surfactant. In a typical procedure, 45 ml of solution-I was obtained by dissolving 15 ml of deionized water in 30 ml of iso-propanol and stirring continuously at 80 °C. Then, solution-II was obtained by dripping 30 ml of titanium tetra iso-propoxide (TTIP) in solution-I under continuous stirring at 80 °C for 1 h. The water-acid mixture (1.5 ml of HCl or HNO_3_ diluted with 50 ml of deionized water) was added to solution-II under stirring. The temperature was reduced from 80 to 60 °C to obtain solution-III. This solution was stirred continuously at 60 °C to obtain white thick precipitated solution, which turned into a transparent white sol after 3 h. To complete the process of hydrolysis and condensation, the sol was stirred further for 150 min. The resultant gel was annealed for 2 h at 300 °C and grinded into fine powder of TiO_2_^12^.

The synthesized TiO_2_ powder was characterized for its surface morphology, particle size, crystallographic phases and band gap energy. The surface morphology was analyzed from SEM images of the sample, particle size and crystallographic phases were analyzed from XRD spectrum and band gap energy was determined from UV–visible spectrum of the sample. After characterizing the photocatalyst, it was used in degrading the pollutants in water under atmospheric plasma exposure.

### Plasma treatment setup

**Figure 1:**
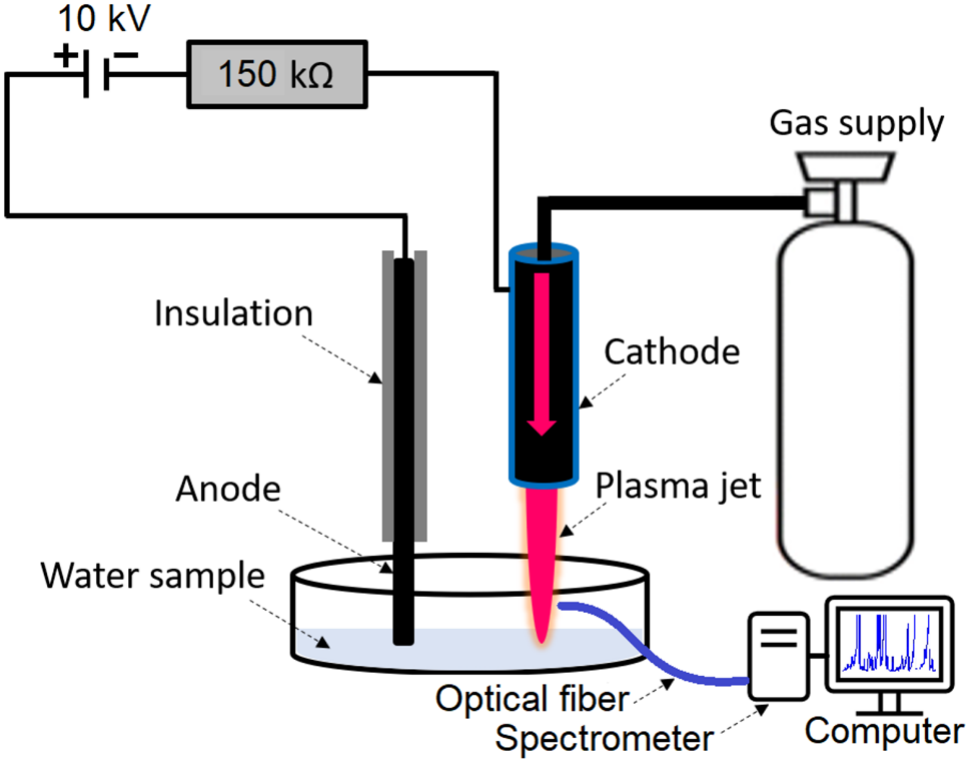
Schematic of DC plasma jet and associated optical emission spectroscopy diagnostic.

A plasma jet was sustained with DC voltage by flowing argon gas through open air and characterized for its chemical composition by using an optical emission spectroscopy technique. Figure 1 shows a schematic of DC plasma jet and associated optical emission spectroscopy diagnostic. The plasma jet is formed of a copper nozzle cathode. The diameter and length of the nozzle is 2 mm and 50 mm, respectively. A 3 mm thick tungsten wire was used as an anode, which was in direct contact with water sample. The cathode was fixed above the water container with a small gap of 10 mm. The gap between electrodes was measured about 30 mm. A DC voltage of 10 kV was applied between cathode and anode. A blast resistance of 150 k Ω was added between cathode and negative terminal of the battery. This safety resistance is used to avoid the breakdown and short circuiting between electrodes during treatment process. An argon-air plasma jet was produced in open atmosphere between cathode and water by supplying argon gas through cathode capillary. This plasma jet interacted with water and transferred the reactive species to the water.

### Plasma–water interaction

The plasma jet may contain positive ions, negative ions, electrons, neutral species and excited species, depending on the environmental conditions. In addition, the plasma jet also releases ultraviolet radiations, which derive the photocatalytic activity of TiO_2_ catalyst. The anticipated plasma jet composition was Oxygen, Nitrogen, Argon ions, excited Argon atoms, neutral Argon atoms, carbon dioxide, nitric oxide, nitrogen dioxide and ozone. The removal of contaminants from water and degradation of organic pollutants takes place when reactive plasma species interact with water body. The oxidation potentials of major reactive radicals are given in Table 1. Optical emission spectroscopy (OES) of the jet was performed to check the presence of reactive species. An optical fiber was used to collect the jet glow signal and to transmit to an Ocean Optic Spectrometer. The spectrometer was attached to a computer where optical emission spectrum of the plasma jet was recorded and analyzed. The light signal was collimated and focused onto the detector where the light signal was converted into an electrical signal. The electrical signal was recorded in the form of a spectrum. The spectrum was recorded in the range of 300–750 nm with an optical resolution of 0.025 nm.

**Table 1:** Oxidation potentials of major reactive radicals or oxidizers.

Oxidizer	Oxidation potential (V)
O^·^	2.42
Ozone (O_3_)	2.07
Hydroxyl radical (OH)	2.80
Hydrogen peroxide (H_2_O_2_)	1.77
Permanganate ion	1.67
Chlorine	1.36
Chlorine oxide	1.50

After OES of the jet, it was used to process the wastewater samples. The water samples were collected from different sites and sources. The details of water sampling are provided in Table 2. In first step, all the water samples were simply plasma treated for 10 min and filtered. In second step, the water samples were treated for the same period in the presence of TiO_2_ catalyst. The catalyst was added to degrade the dyes in the sample under plasma exposure. The treated water was filtered and the residue was analyzed for its chemical composition. The quality of untreated and treated water samples was checked by determining TDS value, pH, conductivity, turbidity, color, etc. The reactive species and ultraviolet radiations in plasma can also kill the bacteria in water during treatment. To check the antibacterial activity of the plasma, the gram-negative and gram-positive bacteria cultures were treated with the jet for 5 min. The colony forming units of bacterial after plasma treatment were counted to study the antibacterial activity of the reactive plasma species.

**Table 2:** Labeling of untreated, plasma treated and plasma/catalyst treated water samples.

Sample labeling before treatment	Sample labeling after plasma treatment	Sample labeling after plasma/catalyst treatment	Sample source
X_1_, X_2_ and X_3_	Y_1_, Y_2_ and Y_3_	Z_1_, Z_2_ and Z_3_	Tap water collected from different sites
X_4_	Y_4_	Z_4_	Effluent of textile industry which contain dyes
X_5_	Y_5_	Z_5_	Mixed wastewater of chemical industry
X_6_	Y_6_	Z_6_	River water
X_7_	Y_7_	Z_7_	Wasted water of filtration plant

### Ethical approval

This article does not contain any studies with human participants or animals performed by any of the authors.

## Results and discussion

### Characteristics of TiO_2_ catalyst

**Figure 2:**
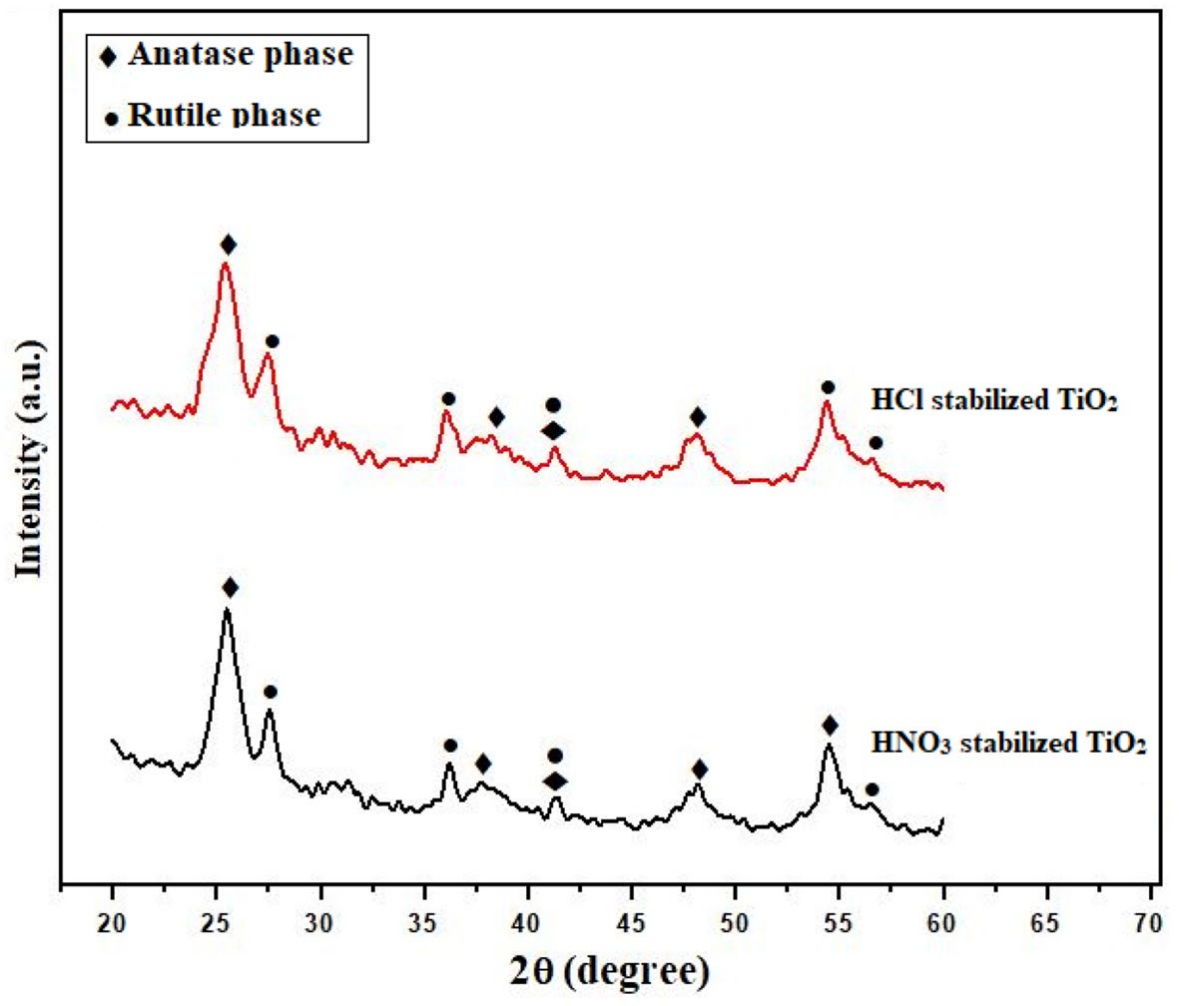
XRD patterns of TiO_2_ nanoparticles stabilized with HCl and HNO_3_ surfactants.

Figure 2 shows XRD patterns of TiO_2_ nanoparticles. The catalyst nanoparticles were composed of mixed anatase and rutile phases. The planes (101), (004), (020) and (121) of anatase phase were identified at 2θ of 25.5°, 38°, 48°and 54°, respectively. Similarly, XRD peaks at 2θ of 27.5°, 36° and 56° correspond to (110), (101) and (220) planes of rutile phase of TiO_2_. The plane (111) at 2θ of 42° reveals both anatase and rutile phases^15,16^. XRD pattern confirmed the crystalline nature of the nanoparticles. The particle size of the synthesized catalyst was determined using the Scherrer’s formula:

1 $$S=\frac{\lambda K}{\mathrm{\beta cos}\theta }$$

where, S is the particle size, K is shape factor or Scherrer constant and is usually equals to 0.89 for spherical shape, λ is the wavelength of X-rays, β is known as full width at half maximum height and *θ* is known as Bragg’s angle.

**Figure 3:**
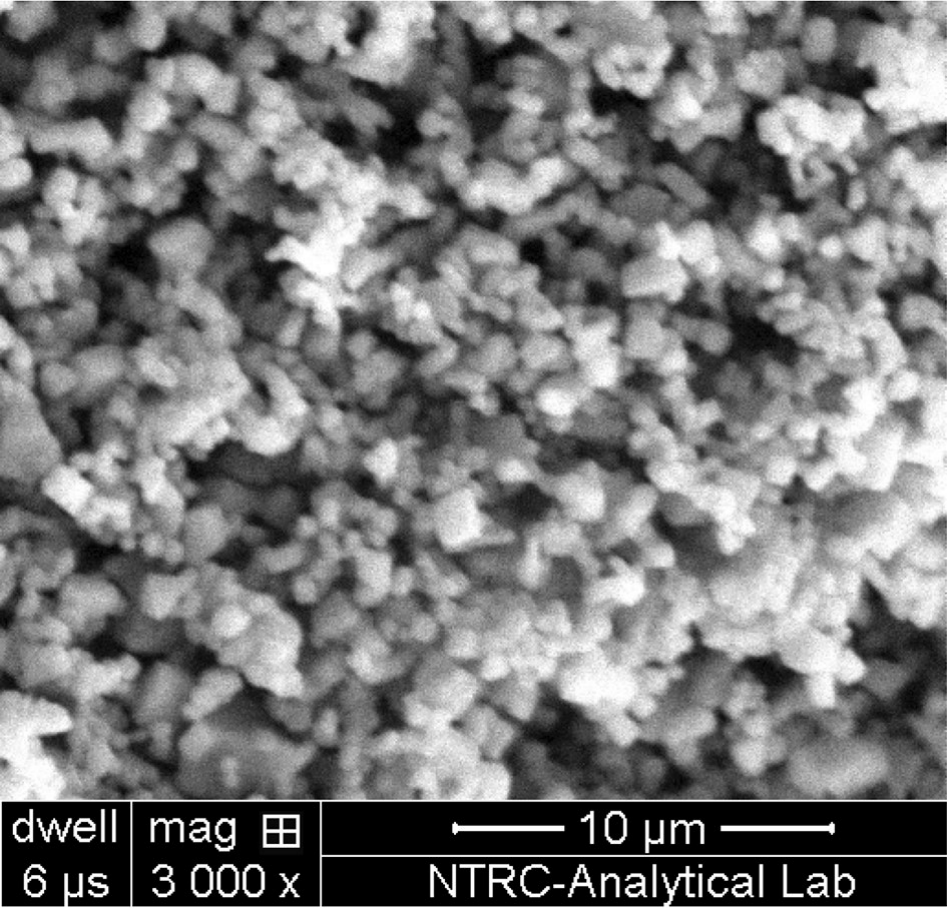
SEM micrograph of TiO_2_ nanoparticles.

The particle size of catalyst varied from 5.2 to 8.5 nm. The particle size of HNO_3_ stabilized nanoparticles remained slightly smaller than HCl stabilized nanoparticles. The surfactants found to be ineffective on phase transformation of TiO_2_ nanoparticles, which mainly depends on the heat treatment^12,17^. The morphology of TiO_2_ nanoparticles was assessed through scanning electron microscopy. The agglomerated spherical nanoparticles were observed in SEM images. Figure 3 shows a typical SEM image of TiO_2_ nanoparticles. In some cases, the formation of agglomerates expands the boundaries between the nanoparticles by changing their shape and size^18,19^.

**Figure 4:**
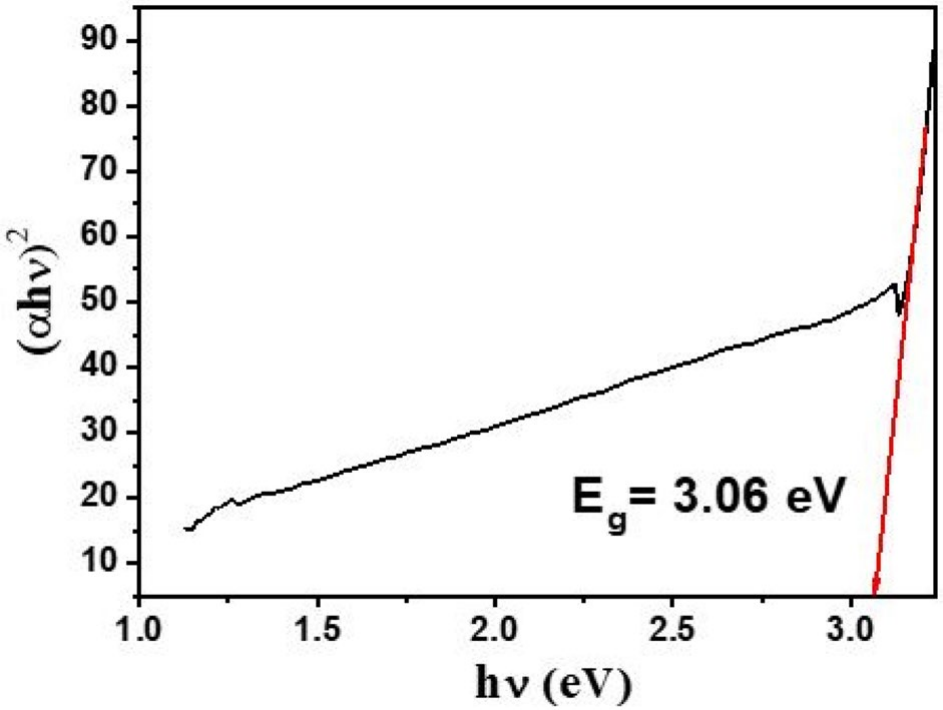
The Tauc-Plot of TiO_2_ nanoparticles for determination of band gap energy.

The band gap energy of TiO_2_ catalyst was determined using Kubelka–Munk equation and UV–visible spectrum of the catalyst^20,21^. The Tauc-Plot of the nanoparticles is shown in Fig. 4. The band gap energy of the nanoparticles was measured about 3.06 eV. The band gap energy of nanoparticles depends on particles^22^. The particle size dependent band gap energy of the catalyst is summarized in Table 3. The band gap energy of the catalyst decreased with an increase in particle size. The change in band gap energy might also be due to phase transformations (i.e. amorphous–anatase–rutile) or induction of charge from bulk to nanocrystals' surface^23,24^.

**Table 3:** Particle size dependent band gap energy of the synthesized TiO_2_ samples.

Particle size (nm)	Band gap energy (eV)
6.4	3.12
5.2	3.18
6.8	3.11
8.1	3.02
7.4	3.06
8.5	2.96

### Optical emission spectroscopy of plasma jet

**Figure 5:**
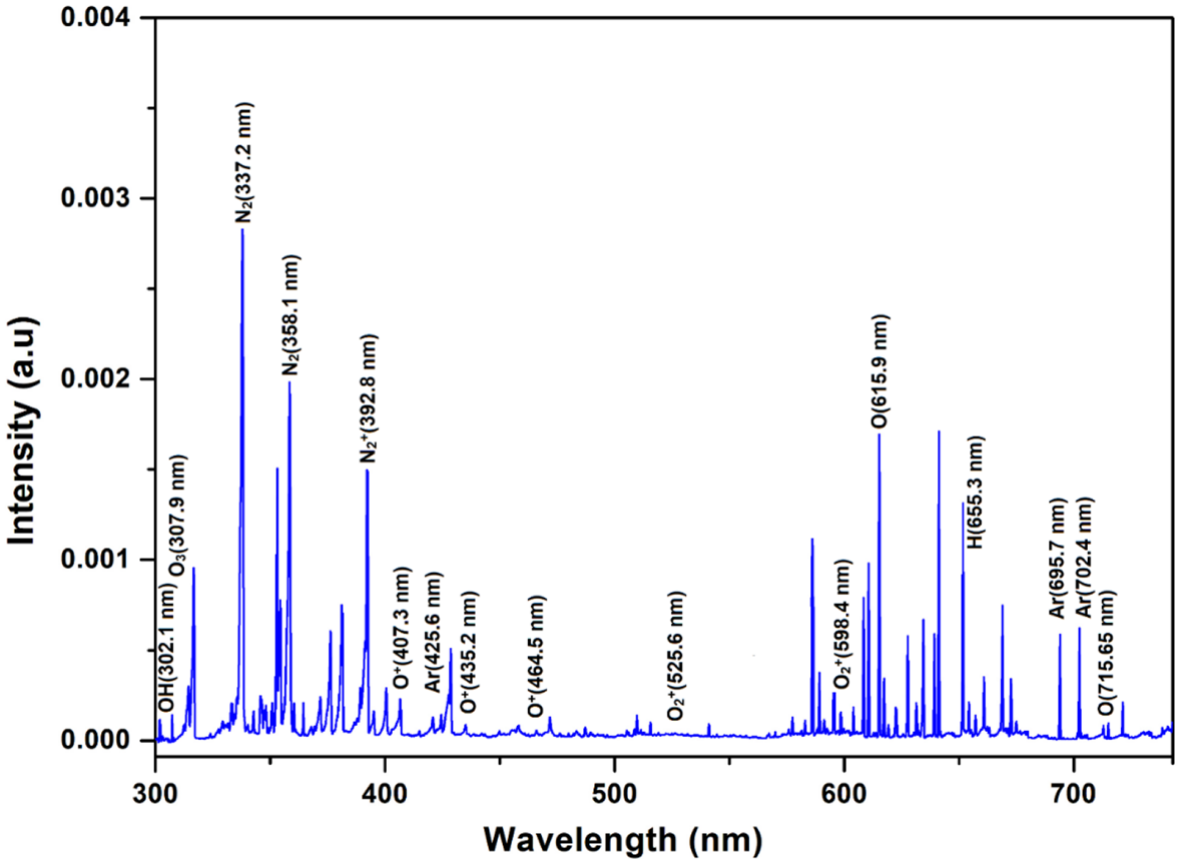
Optical emission spectrum of argon plasma jet in open atmosphere.

Figure 5 shows a typical optical emission spectrum of argon plasma jet having OH, excited nitrogen and oxygen radicals from the air^25^. The emission spectrum confirmed the presence of excited argon, OH, excited nitrogen, excited oxygen, ozone and nitric oxide in the plasma jet. The energetic electrons in the jet excite and ionize the oxygen and nitrogen from the surrounding air. Oxygen molecules break into atomic oxygen to generate ozone through a three-body reaction. On the other hand, the nitrogen in its ground state gets excited due to multiple collisions with electrons as:

2 $$e + N_{2} \left( {X^{1} \sum_{g}^{ + } } \right)\upsilon = 0 \to N_{2} \left( {C^{3} \Pi_{u} } \right)\upsilon^{\prime} = 0 + e$$

The ${N}_{2}({C}^{3}{\Pi }_{u})$ state gets populated through electron impact excitation of ${N}_{2}({X}^{1}{\sum }_{g}^{+})$ ground state and ${N}_{2}({A}^{3}{\sum }_{u}^{+})$ metastable state. Other than electron impact excitation, the associate excitation, penning excitation, pooling reactions and transfer of energy among the colliding particles also populate ${N}_{2}({C}^{3}{\Pi }_{u})$ state^25^. This excited state decays into second positive system of nitrogen by emitting a characteristic photon of (0–0) band^6^ as:

3 $${N}_{2}({C}^{3}{\Pi }_{u}){\upsilon }^{^{\prime}}\to {N}_{2}({B}^{3}{\Pi }_{g}){\upsilon }^{^{\prime}}=o+h\upsilon$$

The second positive system reacts with oxygen molecules to form oxygen radicals, nitrous oxide and ozone. Again, the excited ${N}_{2}^{+}({B}^{2}{\sum }_{u}^{+})$ state of nitrogen gets populated during direct impact ionization of nitrogen in the ground state ${N}_{2}({X}^{1}{\sum }_{g}^{+})$. The populated excited state decays into a first negative system by emitting characteristic photon of (0–0) band. The intensity of the emitted radiations is always proportional to the population density of the excited state.

**Figure 6:**
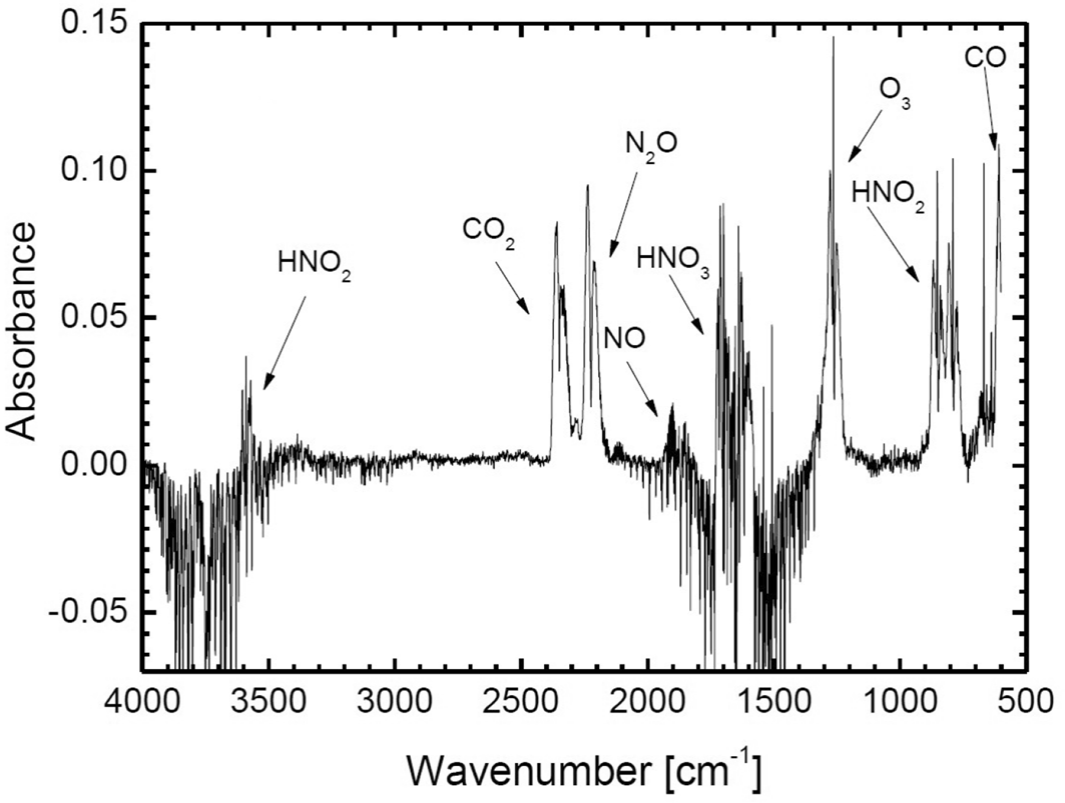
FTIR spectrum of plasma jet during interaction with water in ambient air^27^.

The presence of excited Ar, NO, O_3_, OH^−^, N_2_, ${\mathrm{N}}_{2}^{+}$, O, ${\mathrm{O}}_{2}^{+}$ and O^+^ species in the open atmospheric plasma jet was confirmed from the emission line intensities in optical spectrum at 695–740 nm, 254.3 nm, 307.9 nm, 302–310 nm, 330–380 nm, 390–415 nm, 715.6 nm, 500–600 nm and 400–500 nm, respectively^26^. The emission intensities ratios of the identified species and the second positive system of the nitrogen were found higher in the beginning of the plasma jet excitation. It reveals that the surrounding air quickly diffuses into the jet and the nitrogen concentration increases along the jet flow. Sretenović^27^ characterized the free expanding plasma jet in an open atmosphere. The plasma jet was impinged onto the water surface and characterized for chemical species by generating FTIR spectra at the water-plasma interface. Figure 6 shows a typical FTIR spectrum they produced during water-plasma interaction in ambient air. FTIR absorption detection confirmed the presence of NO, N_2_O, NO_2_, HNO_3_ and HNO_2_ reactive species in the plasma exposed water. The formation of ozone was also noticed during water-plasma interaction both in ambient air and in nitrogen rich environment.

### Water quality parameters

The water quality was checked by determining TDS, pH, conductivity, hardness and color of the samples. Table 4 shows that pH of the water samples noticeably decreased after plasma treatment with and without using a catalyst. The catalyst did not show significant effect on pH of water during plasma treatment. The possible reduction in pH of treated water is referred to the formation of HNO_2_, HNO_3_ and other active ions during water-plasma interaction. The hydrogen ion concentration in water increased with a decrease in pH and so does the water conductivity. Since conductivity depends on concentration of all the active ions present in the sample, pH by itself did not specify the water conductivity. Therefore, pH of water samples did not provide any information about other active ions affecting the conductivity of water. In fact, all the ions in the sample contribute to conductivity. The faster the ions travel towards the opposite electrodes, more conductivity they lead to. The electrical conductivity of treated water samples may also be affected by the type of intermediates formed during plasma-water interaction.

**Table 4:** Water quality parameters of untreated, plasma treated and plasma/catalyst treated water samples.

Sample	pH	Conductivity (µS/cm)	TDS (mg/l)	Hardness (mg/l)	Sulfates (mg/l)	Phosphates (mg/l)
X_1_	8.3	2007	1030	58	59	3.5
Y_1_	7.6	2046	1040	49	48	3.3
Z_1_	7.5	2048	1020	47	40	2.6
X_2_	7.9	831	420	384	54	3.3
Y_2_	7.3	892	420	408	43	2.6
Z_2_	7.4	889	430	400	44	2.9
X_3_	7.9	4660	2370	456	58	2.6
Y_3_	7.5	4728	2370	504	52	2.8
Z_3_	7.3	4739	2390	480	52	3.4
X_4_	12.2	7045	4100	ND	57	2.8
Y_4_	11.6	7971	3960	ND	49	3.3
Z_4_	11.6	7968	3810	ND	50	3.2
X_5_	7.8	1220	620	416	52	3.4
Y_5_	7.3	1252	620	416	49	3.6
Z_5_	7.2	1255	630	440	47	3.3
X_6_	7.9	440	230	184	53	3.5
Y_6_	7.4	474	240	192	50	3.2
Z_6_	7.4	477	240	200	48	3.5
X_7_	7.8	5350	2740	872	48	2.9
Y_7_	7.5	5429	2770	792	44	3.5
Z_7_	7.3	5442	2770	944	41	3.6

The plasma treated samples showed maximum decrease in pH. Most of the untreated water samples were alkaline in nature, which started to neutralize on plasma treatment. There was no significant effect of plasma treatment on the total dissolved solid in water except sample X_4_. This sample exhibited a decrease in TDS by 140 points and 190 points after noncatalytic and catalytic plasma treatment. The hardness of water samples also decreased after plasma treatment. Water with a low pH was less hard, while water with a higher pH was harder or alkaline. The degradation of organic pollutants and dyes in particular increased in the presence of TiO_2_ catalyst and reactive plasma species like Ar, NO, O_3_, OH, N_2_, ${\mathrm{N}}_{2}^{+}$, O, ${\mathrm{O}}_{2}^{+}$ and O^+^. The presence of these reactive species was confirmed through optical emission spectroscopy. Some sulfates and phosphates were also detected in the water samples. The sulfate ions in water samples reacted with plasma generated OH radicals. The degradation of organic pollutants decreases with a decrease in availability of OH radicals. It reveals that for complete degradation of all organic pollutants, prolonged plasma treatment will be needed. Ghezzr et al.^28^ reported that degradation of pollutants in water starts after 20 min of treatment time. The pollutants’ degradation efficiency was measured about 95% after 60 min of noncatalytic plasma treatment. In the presence of TiO_2_ catalyst, the same degradation efficiency was possible only after 30 min of treatment time. It is worth noting that the treatment time mainly depends on plasma intensity and the population of the reactive species. The plasma treatment also cause mineralization of water samples due to formation of chloride ions, sulphate ions and phosphate ions.

Hu et al.^29^ performed photocatalytic decomposition of dyes with TiO_2_ catalyst. The role of inorganic ions in activity of TiO_2_ for dye degradation was investigated. Each dye degraded differently depending on pH of the solution. The sulfate and phosphate ions in the water showed significant effect on dye degradation process. Ghezzar et al.^28^ treated textile wastewaters of different pH values. The wastewater contained azo dyes. A gliding arc discharge plasma was used to treat the dye containing textile wastewaters in the presence of TiO_2_ catalyst. They investigated the role of plasma treatment time and catalyst in degradation of azo dyes. The photocatalytic activity of TiO_2_ was reported higher for the water samples of high pH. The degradation efficiency improved with an increase in treatment time.

### Study of heavy metals

**Figure 7:**
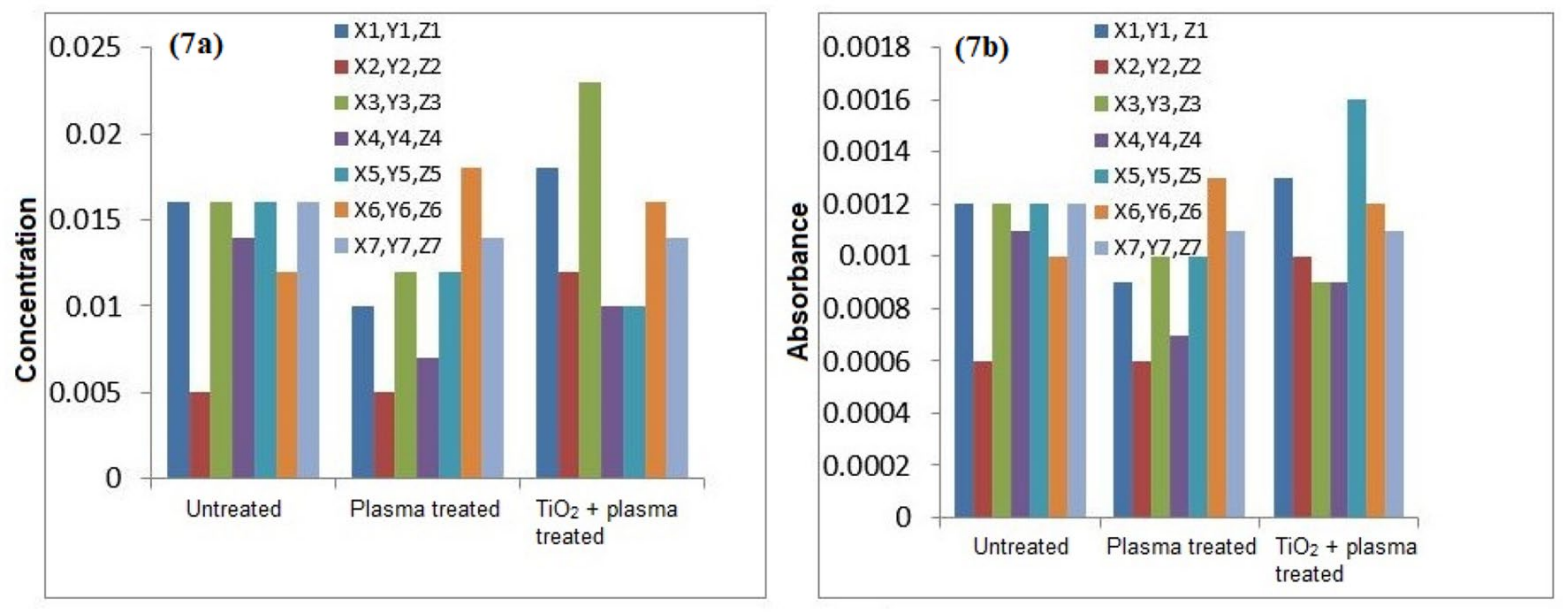
(**a**) Comparison of Cr concentration (mg/l), (**b**) Comparison of Cr absorbance (mg/l).

**Figure 8:**
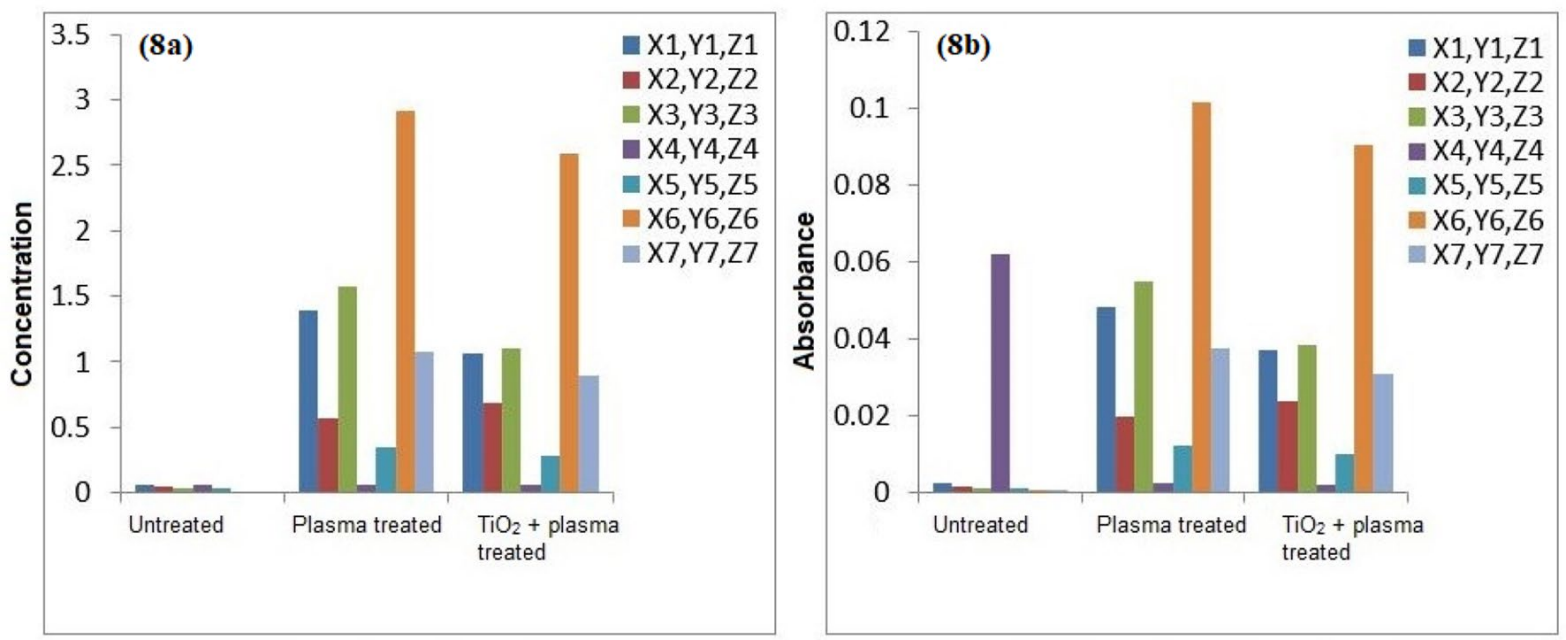
(**a**) Comparison of Cu concentration (mg/l), (**b**) Comparison of Cu absorbance (mg/l).

**Figure 9:**
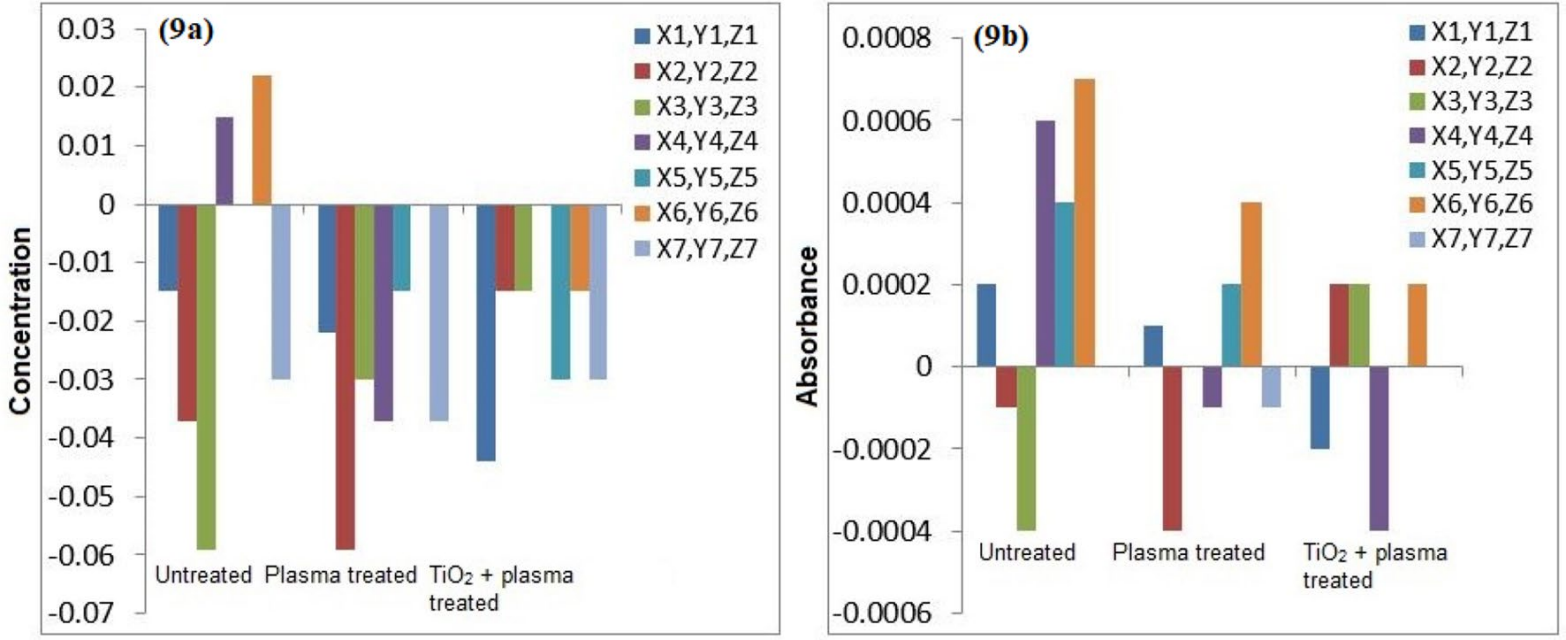
(**a**) Comparison of Pb concentration (mg/l), (**b**) Comparison of Pb absorbance (mg/l).

**Figure 10:**
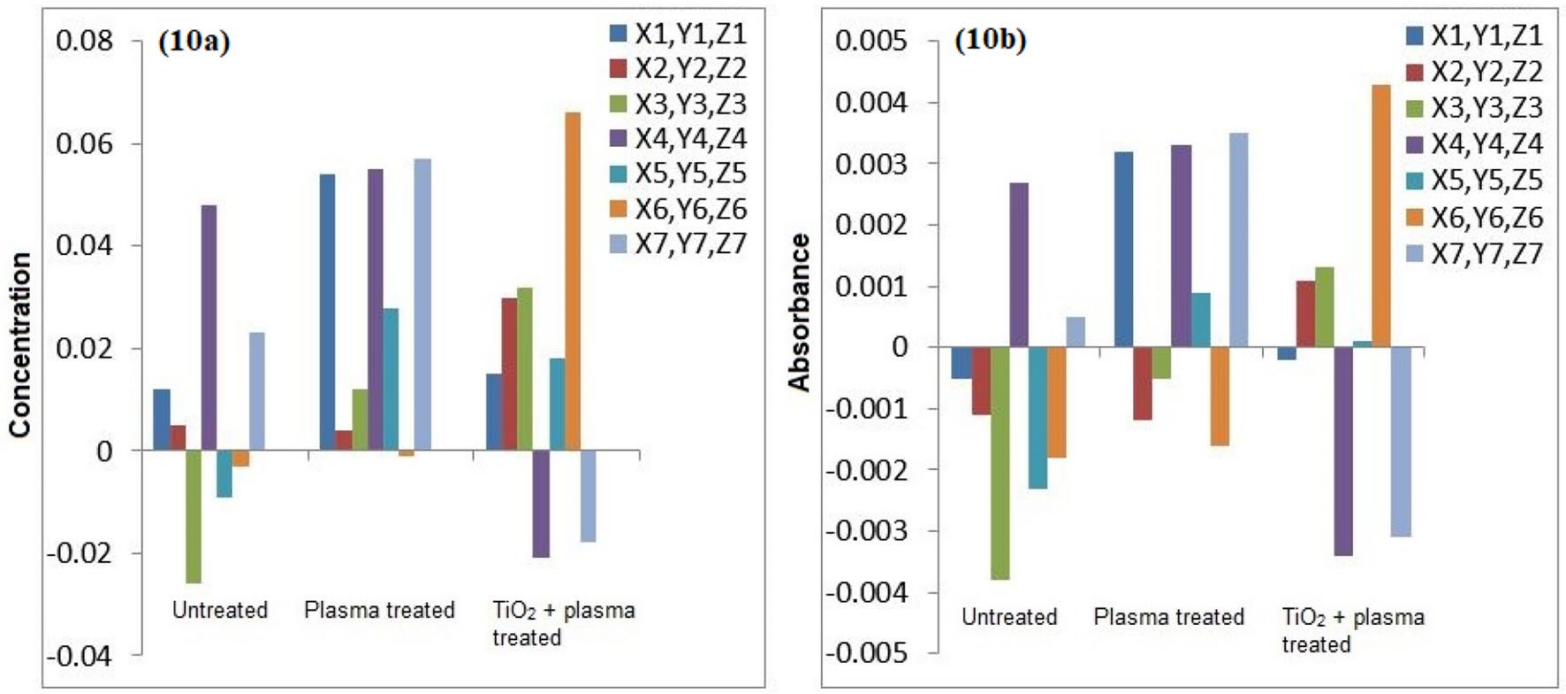
(**a**) Comparison of Ni concentration (mg/l), (**b**) Comparison of Ni absorbance (mg/l).

**Figure 11:**
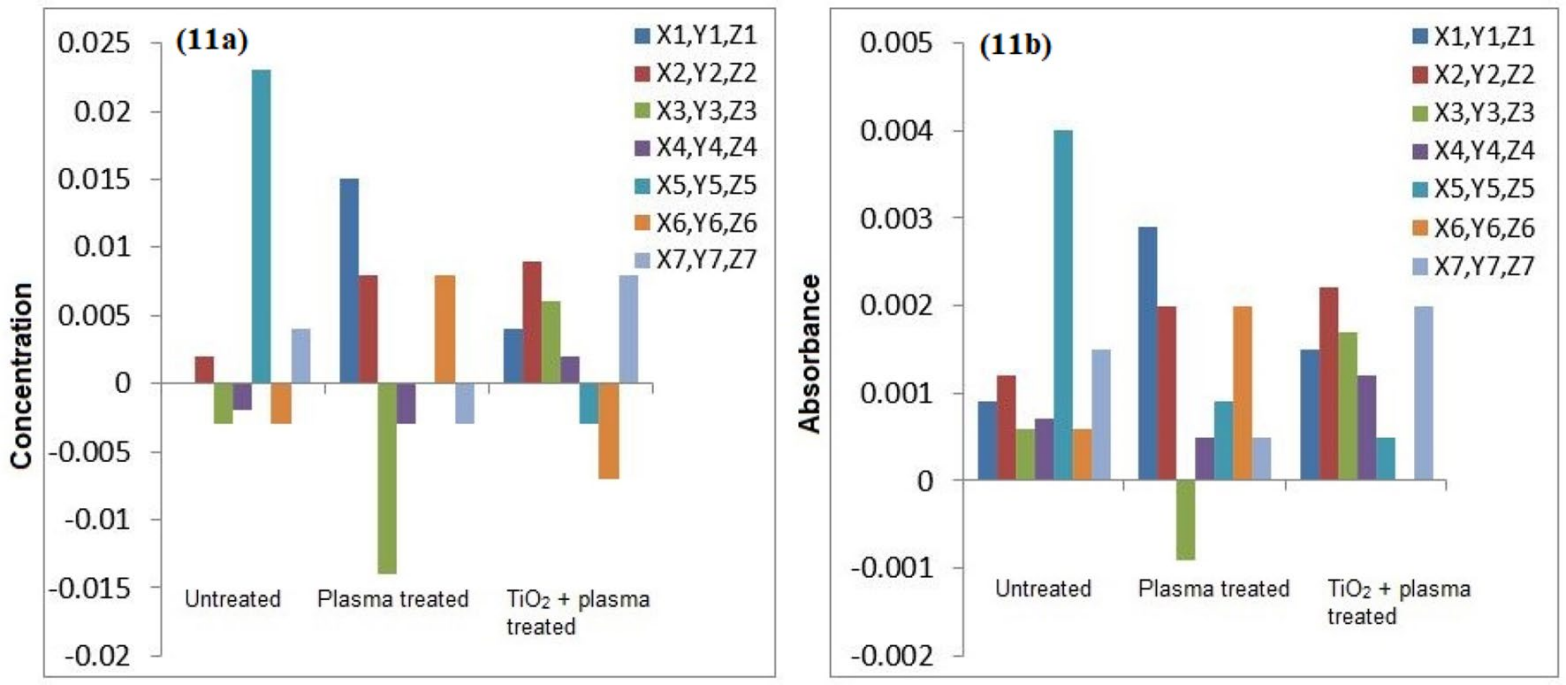
(**a**) Comparison of Cd concentration (mg/l), (**b**) Comparison of Cd absorbance (mg/l).

The atomic absorption spectrophotometry of the untreated and plasma treated water samples was conducted for detection of heavy metals. Figures 7, 8, 9, 10 and 11 confirm the presence of Ni, Cd, Pb, Cr and Cu in the wastewater. Significant amount of heavy metals was detected in the samples. The heavy metals’ content increased on plasma treatment due to mineralization of water samples. Some chloride ions, sulphate ions and phosphate ions also form during plasma treatment. Initially, the water samples were slightly alkaline, which started to neutralize on plasma treatment. Ke et al.^30^ revealed that removal of heavy metals from wastewater is pH dependent. They used argon plasma discharge for removal of chromium through reduction process at plasma–water interface. The reduction efficiency was found higher for solutions with initial pH less than 2 or greater than 8. The reduction efficiency increased on addition of ethanol in the solution. The high reduction efficiency promotes the removal of heavy metals from plasma exposed solution in the form of sediments.

As shown in Fig. 8, the removal of Cu in plasma and plasma/TiO_2_ treated water samples was found higher than the untreated water. The removed metals settle at the bottom, which were removed through filtration. The residue of untreated water contained negligible amount of Cu. The removal of metals from treated water increases due to the formation of byproducts in the water during plasma exposure. The Pb removal efficiency of plasma treatment was significant higher. After plasma treatment, Pb was not detected in water samples. Similar trend was predicted for other metals.

Icopini et al.^31^ removed Cr from water samples of different pH values. The metal removal efficiency was reported higher for lower pH values. It was revealed that Cr containing samples would be neutral or positively charged when pH of the sample is low. In the presented work, pH of solution decreases on plasma treatment, which promotes the removal of metals. Cserfalvi et al.^32^ tested an atmospheric gas discharge technique for determination of heavy metals in different solutions. For lower pH values, the sputtering of solution surface during plasma exposure and subsequent excitations within the solution were observed. Using electrolyte-cathode discharge spectrometry technique, they identified Ni, Pb, Cu, Zn, Mn and Cd metals in the aqueous solutions. The emission peak intensity and concentration of these metals depended on pH of solution and hydrogen ion concentration during plasma exposure.

### FTIR and XRD analysis of residue

**Figure 12:**
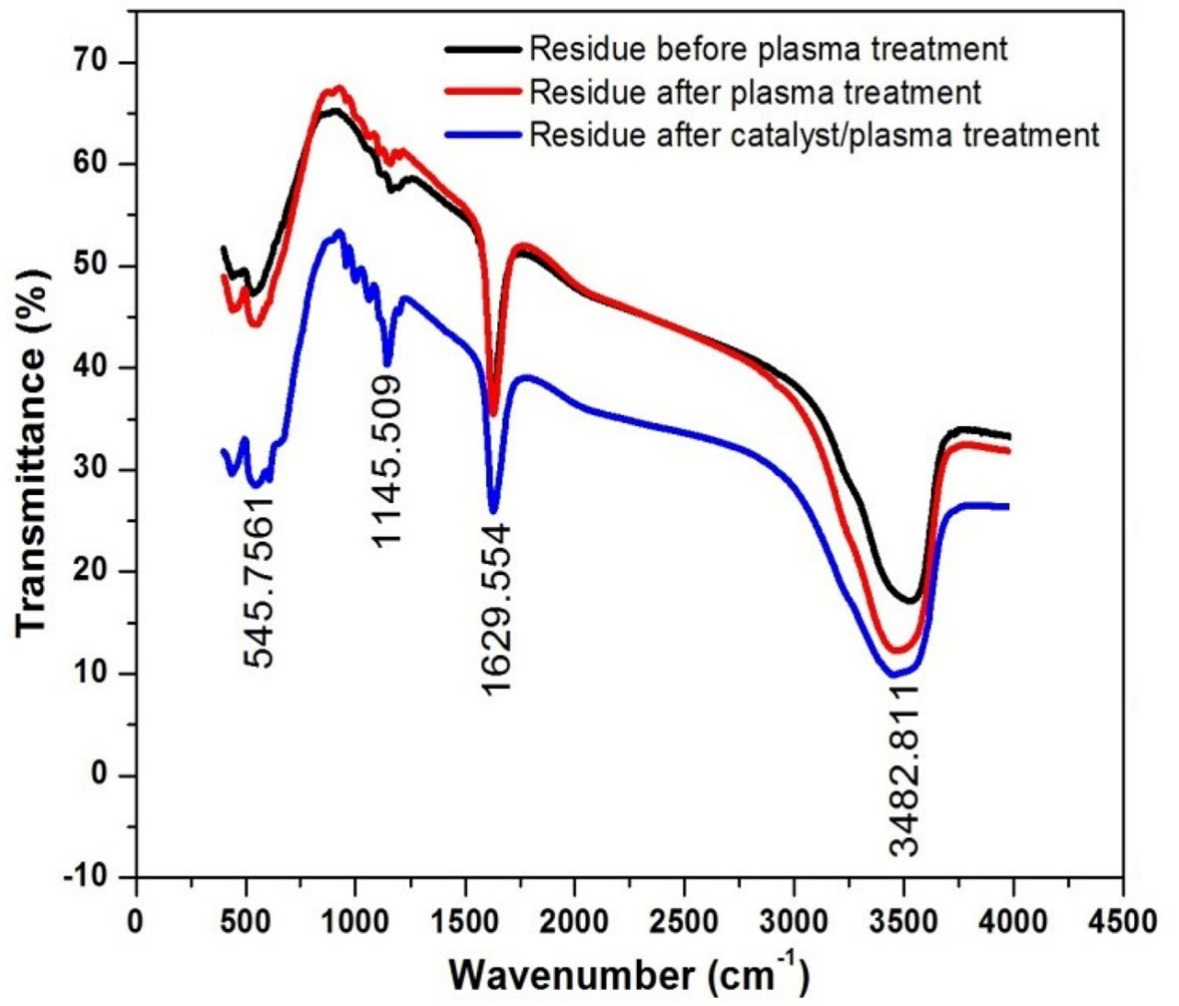
FTIR profiles of solid residues of untreated and treated wastewater.

Figure 12 shows FTIR spectra of untreated and plasma treated samples. FTIR analysis confirmed the presence of amines, hydroxyl groups, amides, esters, ethers, anhydrides and carboxylic acids in the sample. The N–H stretching of primary amines, aromatic amines and amides was observed in the wavenumber range of 3320–3520 cm^−1^. Eithers with C–O–C linkage were observed in the wavenumber range of 1070–1240 cm^−1^. Sulfates had SO_2_ symmetric stretching in the wavenumber range of 1140–1200 cm^−1^. Similarly, ketones with C–C=O group were identified in the wavenumber of 510–560 cm^−1^. The reported results were inline with the findings of Tichonovas et al.^8^. They treated polluted water samples with a barrier discharge system. The plasma treated samples contained amides, amines, carboxylic acids and nitrates.

**Figure 13:**
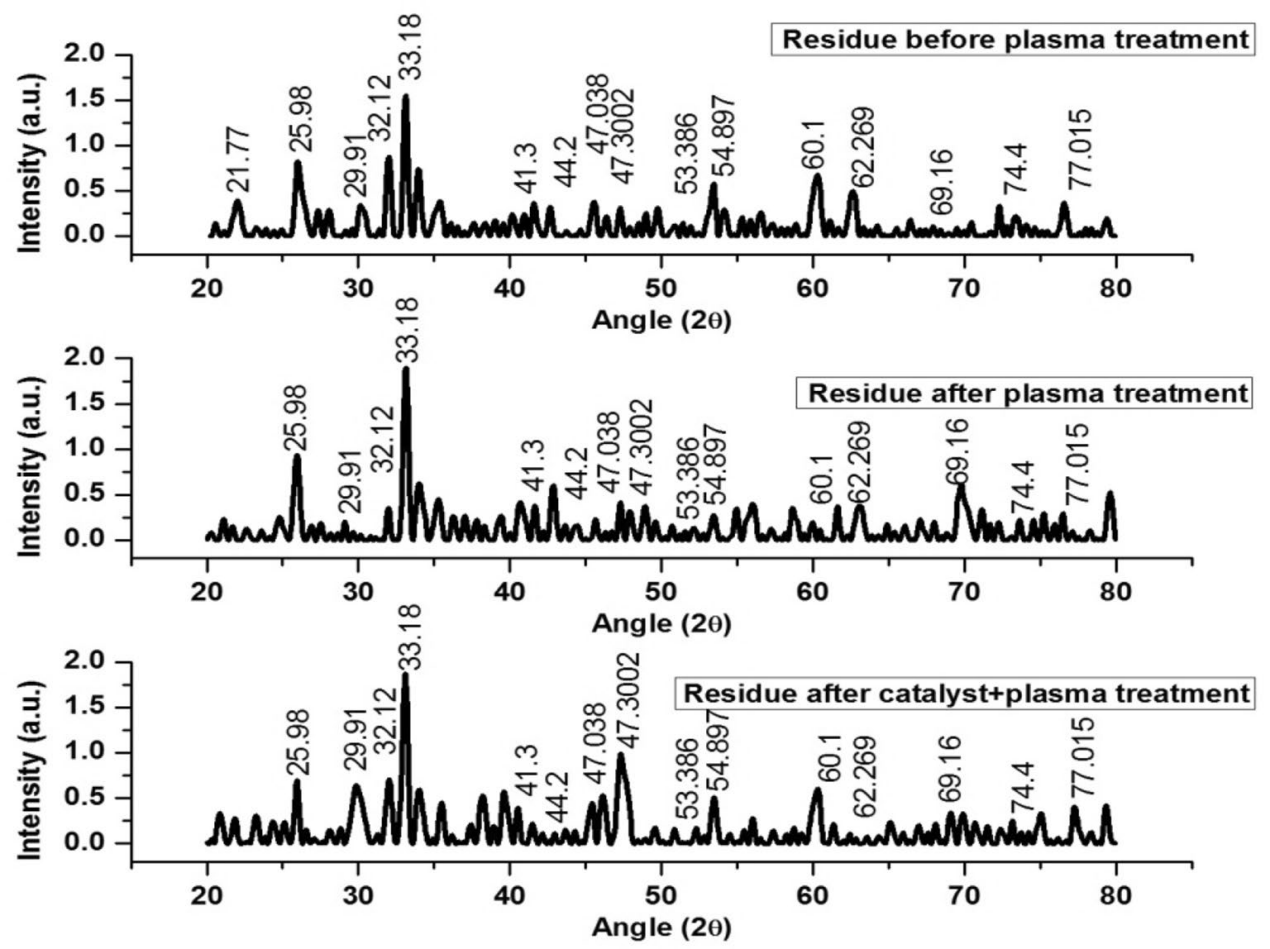
XRD profiles of solid residues of untreated and treated wastewater.

The water samples were filtered to remove the solid residue. The residue was characterized for its chemical composition. Figure 13 shows XRD spectra of the residue of untreated and treated wastewater samples. XRD peaks at 2θ of 25.98°, 29.91°, 32.12°, 44.2°, 47.038°, 47.30°, 54.9°, 60.1°, 69.16° and 74.4° correspond to S, Alite (triclinic), ferrite, Ni, CdS, Si, SiO_4_, Ag, Pb, CdO and Cu, respectively. Similarly, XRD peaks at 2θ of 41.3° and 53.386° correspond to Cr_3_O_4_, 21.77° and 33.18° correspond to Aluminate, and 62.269° and 77.015° correspond to Pb. The peak at 47.3002 shows the presence of 220 plane of Si. Alite, ferrite and aluminate had similar peaks as described somewhere else^33^. Sharma et al.^33^ treated polluted water with metallic nanoparticles. The nanoparticles were used to remove heavy metals for the wastewater. Several metals were identified and removed from industrial effluents collected from different industrial sites in India.

### Antibacterial activity of plasma species

**Figure 14:**
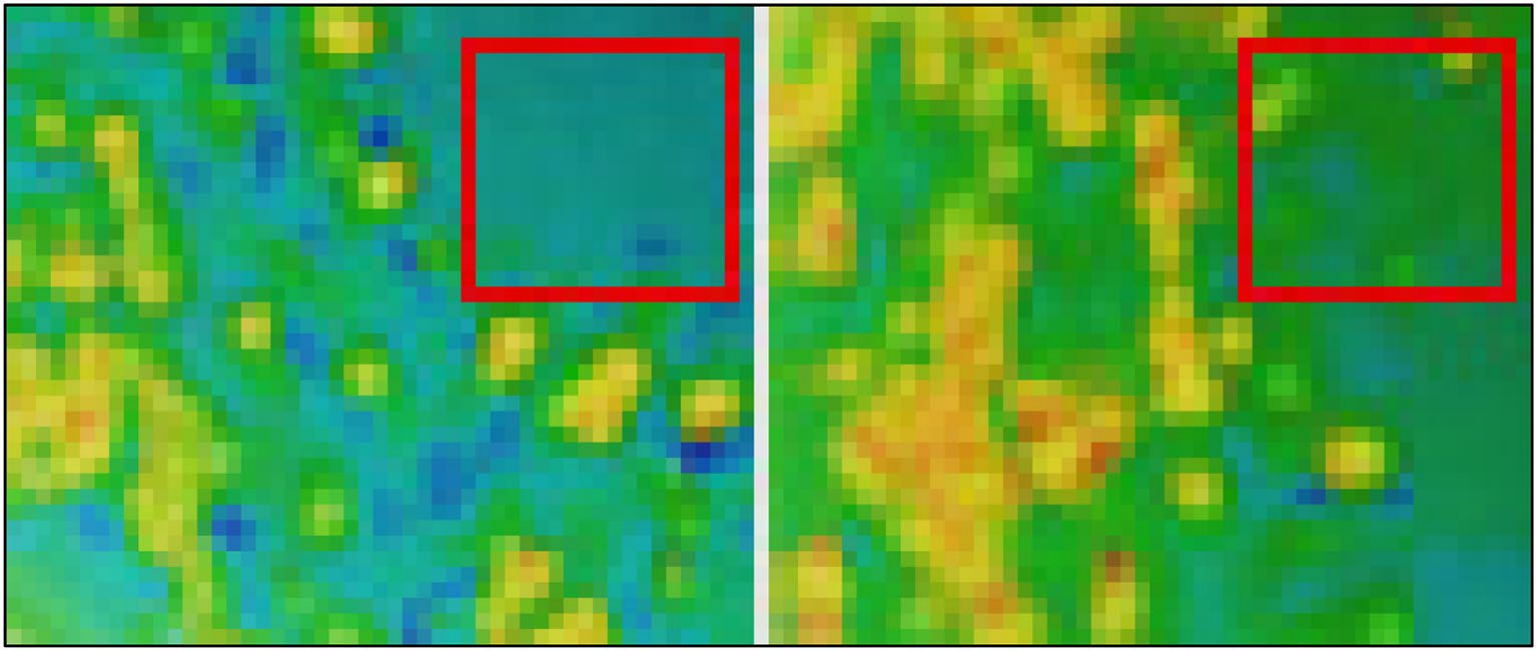
Scattering configuration of bacteria CFUs on the petri dish before and after plasma treatment for 5 min: *Staphylococcus aureus* (left image) and *Escherichia coli* (right image).

As discussed earlier, the plasma jet contained some strong oxidizers, which can easy kill the bacterial endospores and vegetative cells. Other than the plasma born ultraviolet radiations, the ozone, atomic oxygen and free reactive oxygen radicals also damage the cells by charging the cell wall and reacting with macromolecules. Since membrane lipids at the cell surface are susceptible to the reactive oxygen species, the oxidized cytoplasmic membrane lipids release intracellular substances, which damage the cells. The ultraviolet radiations interact with spore surface and cause volatilization of the surface compounds and damaging of DNA. The reactive oxygen radicals apply the electrostatic forces by charging the cell wall and oxidize the spore surface. In this study, the effect of plasma on inactivation of different bacteria in water was investigated. The culture of *Escherichia coli* (gram positive) and *Staphylococcus aureus* (gram negative) was subjected to the plasma exposure. The efficacy of plasma treatment to inactivate the bacteria was determined by observing colony forming unit (CFU) counts before and after plasma exposure. Figure 14 shows photographically the plasma exposed regions of the bacterial culture. The plasma treated regions are marked with square boundaries. A colony counter was used to find the CFU/plate. Significant reduction in CFU was observed after plasma exposure. Roughly, 98% decay of both cultures was observed after treatment time of 5 min.

Initially, without any plasma exposure, the effect of air on bacteria deactivation was observed. The air flow did not show any effect on bacterial CFU. Thereafter, bacteria cultures were exposed to plasma and CFUs were counted before and after plasma exposure in the marked area of the petri dish. Since bacteria have several protective layers surrounding the genetic nucleus, it was difficult to kill them in the unexposed areas. However, all the bacterial were dead in the areas directly exposed to plasma. For prolonged plasma exposure, the bacteria in the adjacent regions also started to deactivate. The effect of plasma treatment on Staphylococcus aureus was more pronounced than the Escherichia Coli. All the Staphylococcus aureus cells in the plasma exposed region were found dead after 5 min of treatment while some Escherichia Coli cells were still alive in the plasma exposed region. It is possible to neutralize all the cells by increasing the treatment time.

## Conclusions

Catalytic plasma treatment of wastewaters was conducted in ambient air in the presence of TiO_2_ catalyst. The catalyst nanoparticles were composed of mixed anatase and rutile phases with particle size in the range of 5.2–8.5 nm. The optical emission spectroscopy confirmed the presence of excited argon, OH, excited nitrogen, excited oxygen, ozone and nitric oxide in the plasma jet. The energetic electrons in the jet excited and ionized the oxygen and nitrogen from the surrounding air. The spectral lines of Ar, NO, O_3_, OH^−^, N_2_, ${\mathrm{N}}_{2}^{+}$, O, ${\mathrm{O}}_{2}^{+}$ and O^+^ species were observed at wavelength of 695–740 nm, 254.3 nm, 307.9 nm, 302–310 nm, 330–380 nm, 390–415 nm, 715.6 nm, 500–600 nm and 400–500 nm. These reactive plasma species degraded the organic pollutants and separated the heavy metals from the wastewater. The conductivity of the water samples increased while pH and hardness decreased on treatment. The atomic absorption spectrophotometry of the samples confirmed the presence of heavy metals, which were effectively removed through plasma treatment. FTIR analysis confirmed the presence of amines, hydroxyl groups, amides, esters, ethers, anhydrides and carboxylic acids in the samples. XRD analysis of the solid residue confirmed the presence of S, Alite (triclinic), ferrite, Ni, CdS, Si, SiO_4_, Ag, Pb, CdO, Cu, Cr_3_O_4_ and Aluminate in the samples. On the antibacterial side, the effect of plasma treatment on Staphylococcus aureus was more pronounced than the *Escherichia coli*. Overall, 98% decay of both bacterial cultures was observed after plasma treatment for 5 min. These findings confirm that the reported plasma jet technique is effective for degradation of organic pollutants, inactivation of bacterial and separation of inorganic pollutants from the wastewaters.
